# 

**Published:** 1924-10

**Authors:** 


					New Series. Vol.III. No. 37^^?). October, 1924. Monthly, Price 6d. (ros7a5REE)

				

